# Feasibility, acceptability, and efficacy of online supportive care for individuals living with and beyond lung cancer: a systematic review

**DOI:** 10.1007/s00520-021-06274-x

**Published:** 2021-05-18

**Authors:** Jordan Curry, Michael Patterson, Sarah Greenley, Mark Pearson, Cynthia C. Forbes

**Affiliations:** 1 Wolfson Palliative Care Research Centre, Hull York Medical School, University of Hull, Cottingham Road, Hull, UK; 2 Institute for Clinical and Applied Health Research, Hull York Medical School, University of Hull, Cottingham Road, Hull, UK

**Keywords:** Lung neoplasms, Supportive care, Online, Feasibility, Review

## Abstract

**Purpose:**

To examine the evidence of the feasibility, acceptability, and potential efficacy of online supportive care interventions for people living with and beyond lung cancer (LWBLC).

**Methods:**

Studies were identified through searches of Medline, EMBASE, PsychINFO, and CINAHL databases using a structured search strategy. The inclusion criteria (1) examined the feasibility, acceptability, and/or efficacy of an online intervention aiming to provide supportive care for people living with and beyond lung cancer; (2) delivered an intervention in a single arm or RCT study pre/post design; (3) if a mixed sample, presented independent lung cancer data.

**Results:**

Eight studies were included; two randomised controlled trials (RCTs). Included studies reported on the following outcomes: feasibility and acceptability of an online, supportive care intervention, and/or changes in quality of life, emotional functioning, physical functioning, and/or symptom distress.

**Conclusion:**

Preliminary evidence suggests that online supportive care among individuals LWBLC is feasible and acceptable, although there is little high-level evidence. Most were small pilot and feasibility studies, suggesting that online supportive care in this group is in its infancy. The integration of online supportive care into the cancer pathway may improve quality of life, physical and emotional functioning, and reduce symptom distress. Online modalities of supportive care can increase reach and accessibility of supportive care platforms, which could provide tailored support. People LWBLC display high symptom burden and unmet supportive care needs. More research is needed to address the dearth of literature in online supportive care for people LWBLC.

**Supplementary Information:**

The online version contains supplementary material available at 10.1007/s00520-021-06274-x.

## Introduction

Lung cancer is the leading cause of cancer-related death internationally for both men and women [1]. It is a debilitating disease which has a large effect on quality of life (QoL) [1]. Though the median life expectancy for people diagnosed with lung cancer remains poor, advances in screening and curative treatments for lung cancer have contributed to the 9% reduction in mortality over the last decade and extended life expectancy [2, 3]. Increasing survival rates have been reported [4, 5], however, curative treatments can elicit a myriad of adverse physiological and psychological effects which can reduce QoL (e.g. fatigue, dyspnea, and depression) [1, 6, 7]. In fact, people living with and beyond lung cancer (LWBLC) have reported greater unmet psychological and physiological needs in comparison to other types of cancer [8, 9]. Unmet needs are those needs which do meet the level of support required for optimal health [10].

Supportive care can be defined as care that helps an individual living with and beyond cancer and/or their immediate family or caregivers cope throughout the treatment pathway, from diagnosis to continuation through the illness or death [5]. Evidence suggests that supportive care needs for people LWBLC have noticeably increased [11]. A systematic review examining the supportive care needs of people living with lung cancer reported nine distinct domains of supportive care needs: physical, psychological, spiritual, cognitive, communication, social, daily living, practical, and informational [12]. The nine domains highlight the considerable burden among people LWBLC and the importance of supportive care interventions.

The internet and digital technology have become an important resource used within the oncology community, both for people living with and beyond cancer and oncology professionals [3]. Utilizing digital technology to deliver oncological supportive care has attracted significant interest over the recent years [13–15], with the potential to deliver tailored, inexpensive care while achieving mass reach [16, 17].

Thus far, online health and supportive care services have largely focused on breast and prostate cancer [17, 18]. Few online supportive care platforms exist for people LWBLC, despite the need and potential benefits to patients [13–15]. Though reviews have explored the use of online and digital interventions among mixed cancer types [17, 19], specific cancer-related needs and symptom burden vary considerably [17]. Those who live five or more years post-lung cancer diagnosis are referred to as ‘Long-Term Lung Cancer Survivors’ (LTLCS) [20]. In comparison to their age-matched counterparts from other types of long-term cancer survivors, LTLCS display the lowest QoL [20, 21]. In the USA, an estimated one in four LTLCS are living with significant restrictions in physical functioning and depressive mood symptoms [20].

McAlpine et al. (2015) critically examined the efficacy of online interventions for cancer patients, highlighting the uncertainty of the benefits with mixed results [17]. Among the 14 studies included in the McAlpine review, the majority focused solely on breast cancer with only three studies independently reporting lung cancer, head and neck cancer, prostate cancer, and four reporting mixed cancer types. McAlpine and colleagues illustrate that though there is increasing interest in online technology within oncology care, there is a lack of literature regarding efficacy. This may be partially due to the small portion of studies which present a quantifiable and a clinically meaningful evidence-base [17].

To appropriately develop and appraise literature for people LWBLC, cancer type must be used as a moderator, allowing specific evaluation on the feasibility, acceptability, and efficacy of online technologies for people LWBLC. Thus, this review aims to examine the evidence of the feasibility, acceptability, and potential efficacy of online supportive care interventions for people LWBLC. For the purpose of this review, online supportive care will be defined as interventions delivered using online mediums which aim to meet a person’s physical, social, informational, spiritual, practical, and/or psychological needs during the diagnostic, treatment, and follow-up phases of the cancer spectrum [22]. This review will examine individuals LWBLC. For the purpose of this study, LWBLC is any individual who has had a diagnosis of lung cancer or cancer within the lungs.

## Methods

The review adheres to the reporting of the Preferred Reporting Items for Systematic Reviews and Meta-Analyses [23]. A standardised data extraction form [24] was adapted for the extraction and review of all data. Ethical approval was not required.

### Eligibility criteria

Eligibility of studies was based upon inclusion and exclusion criteria developed a priori (PROSPERO ID: CRD42020171847). A study’s eligibility was based on whether it met the following conditions: (1) examined the feasibility, acceptability, and/or efficacy of an online intervention aiming to provide supportive care for people LWBLC; (2) single arm or RCT study pre/post design; (3) if a mixed sample, presented independent lung cancer data. Studies were excluded based on the following conditions: (1) mixed sample data was presented with no individual lung cancer data (mixed cancer types); (2) articles were not provided in English; (3) full text articles were not available.

### Search strategy

The databases EMBASE, Medline, PsychINFO via OVID, and CINAHL via EBSCOhost were searched from their inception up to April 2020. MeSH terms were identified for the key concepts in Medline and the equivalent adapted for subsequent databases. The development of the search strategies, per database, was completed with the assistance of an Information Specialist (SG). Boolean operators were used to combine MeSH terms and keyword terms to develop a pilot strategy. The pilot strategy was executed in Medline and refined to ensure the relevance of the search output (see Online Resource 1). The search strategy for Medline, EMBASE, and PsycINFO focused on the following: lung cancer AND (Internet OR social media/online supportive care interventions). Whereas the terms in CINAHL were lung cancer AND social media platforms AND internet platforms. All searches were conducted by a single author (JC).

### Study selection

All articles identified through the database searches were exported to a citation management software (EndNote, X9.2), wherein duplicates were removed. Rayyan citation screening software was used post-deduplication by two authors (JC and MPa) to screen titles and abstracts against pre-specified inclusion criteria. Disagreements were discussed and resolved by mutual consensus.

### Data extraction and methodological quality assessment

Data from the included studies were extracted using a data extraction form, which was developed by the research team following a recommended template [24]. Data regarding study setting, participant characteristics, study design, intervention procedure, outcome results, and findings relating to feasibility, acceptability, and efficacy of the intervention were extracted. The extraction form was piloted by two of the authors (JC and CF) to ensure it captured all relevant information on paper. No changes were made, and the remaining articles were extracted independently by JC, with 100% of the articles also extracted by a second author (MPa). The two authors had one disagreement regarding the extraction of qualitative text from one article [15], but was resolved by mutual consensus with input from a third author (CF).

The methodological quality of the studies was assessed via the Standard Quality Assessment Criteria for Evaluating Primary Research Papers from a Variety of Fields [25]. This tool provides independent subscales for methodological assessment of qualitative and quantitative data. The tool allows for a broad assessment of quantitative studies including non-randomised, pilot, and feasibility studies. The tool selected for this review was with consideration of the study designs [26, 27] and prior literature [28, 29] in mind. The tool was chosen based on the importance of including a wide range of study designs, as it has been noted that within single study designs, aspects such as feasibility, reliability, validity, and utility are variables often unmeasured [30].

Study quality was rated in accordance with the following accepted scoring methods, > 80% “strong”, 71–79% “good”, 50–70% “adequate”, and < 50% “poor” [28, 29, 31]. If any uncertainty surrounding the initial assessment of the level of bias within a study was noted between the two authors, a member of the research team (MPa) assisted in reaching a consensus. Studies were not excluded from the synthesis of this review based on the rating of study quality.

### Outcomes

The following outcomes were assessed to ascertain feasibility: (1) recruitment and retention rates, (2) recruitment barriers, (3) intended implementation, (4) cost of implementation. Outcomes assessing acceptability were: (1) acceptability and satisfaction, (2) intervention adherence rates, (3) intervention burden, (4) noted adverse effects. Efficacy was reported for RCTs only. The outcomes relating to efficacy was assessed by the effect of supportive care relative to the comparison group for the outcome measured.

## Results

### Study selection

**Fig. 1:**
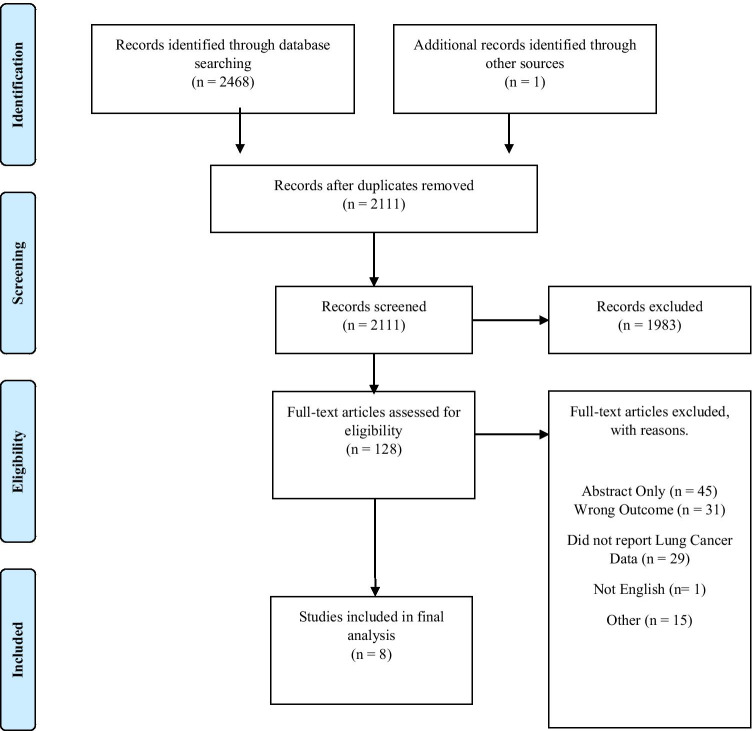
PRISMA flow diagram [23]

A flow chart detailing the study selection process is presented in Fig. 1. A total of 2468 publications were identified from the following databases: Medline, EMBASE, PsycINFO, and CINAHL. One additional article was identified through hand searching. After the removal of duplicates, 2111 articles were included in title and abstract screening and 128 studies were included in full text screening. Finally, eight articles were acknowledged as meeting the eligibility criteria and were included in data extraction.

### Risk of bias/methodological assessment

Findings from the methodological quality assessment are presented for quantitative measures in Table 1 and qualitative measures in Table 2. Based on the assessment conducted independently by two reviewers (JC and CF), six studies were assessed for quantitative methods [32–37] and two studies assessed for both quantitative and qualitative methods [13, 15]. Based on quantitative methods, eight studies were rated as strong [13, 15, 32–37]. For qualitative methods, one study was rated strong [15] and one adequate [13].

**Table 1:** Illustrating the breakdown of quality appraisal scores and inter-rater reliability values for quantitative method studies

Author	Checklist item														Inter-rater reliability
	1) Question or objective sufficiently described?	2) Evident and appropriate design	3) Subject selection	4) Subject characteristics	5) Random allocation	6) Blinding of investigators	7) Blinding of subjects	8) Defined androbust outcome measures	9) Sample size	10) Analysis described and appropriate	11) Estimate of variance	12) Controlled for confounding	13) Sufficient Results	14) Results match Conclusions?	
Huang et al., (2019) [36]	2	2	2	2	2	N/A	N/A	2	2	2	2	0	2	2	1 (100%)
Park et al., (2019) [34]	2	2	2	2	2	N/A	N/A	2	2	2	2	N/A	2	2	1 (100%)
Ji et al., (2019) [33]	2	2	2	2	1	N/A	N/A	2	2	1	2	2	2	2	1 (100%)
Lafaro et al., (2019) [37]	2	1	2	2	N/A	N/A	N/A	1	1	2	1	N/A	2	2	1 (100%)
Timmerman et al., (2017) [13]	2	2	2	1	N/A	N/A	N/A	2	1	2	2	N/A	2	2	1 (100%)
Coats et al., (2019) [35]	2	2	1	2	N/A	N/A	N/A	2	1	2	2	1	2	2	1 (100%)
Maguire et al., (2015) [15]	2	2	2	2	N/A	N/A	N/A	2	2	2	2	N/A	2	2	1 (100%)
Denis et al., (2014) [32]	1	1	2	2	N/A	N/A	N/A	2	1	2	N/A	N/A	2	2	1 (100%)

**Table 2:** Illustrating the breakdown of quality appraisal scores and inter-rater reliability values for qualitative method studies

Author	Checklist item										**Inter-rater reliability**
	1) Question or objective sufficiently described?	2) Evident And appropriate design	3) Clear context for the study	4) Linked to a theoretical framework	5) Appropriate and detailed sampling strategy	6) Clear and detailed data collection methods	7) Complete, appropriate and systematic data analysis	8) Verification procedure(s) used in the study	9) Conclusions supported by results?	10) Evident reflexivity	
Maguire et al., (2015) [15]	2	2	2	2	2	2	2	0	2	0	1 (100%)
Timmerman et al., (2017) [13]	2	2	1	1	1	1	1	2	2	0	1 (100%)

### Study characteristics

This review included two RCTs [33, 36] and six pilot and feasibility studies [13, 15, 32, 34, 35, 37]. The included studies were carried out in seven different countries, (two in South Korea [33, 34], one each in the USA [37], France [32], Canada [35], Netherlands [13], Taiwan [36], and the UK [15]). Of the eight studies, seven comprised solely of individuals LWBLC [13, 15, 32–36], one study explored both carers and individuals living with and beyond gastrointestinal cancer or lung cancer [37]. Specifically, of the studies focusing on independent lung cancer populations, four focused on Non-Small Cell Lung Cancer (NSCLC) [13, 33, 34, 36], one focused on surgical excision [32], one explored patients with lung cancer, receiving a specific course of radiotherapy [15], and unresectable thoracic neoplasia [35].

Five studies reported the cancer disease stage, ranging from I to IVb [32–36], one study reported the ASA Physical Status Classification System (ASA) [37], and two studies did not report the stage of cancer [13, 15]. Treatment types reported were chemotherapy [34–36], thoracic radiotherapy [15], and maintenance therapy [32]. One study reported the extent of surgery participants had [13] and two did not report any treatment information [33, 37]. Further information regarding the study characteristics can be found in Table 3.

**Table 3:** Study characteristics

Study details (author and year)	Population characteristics				Feasibility		
	Sample size; age	Location		Disease stage; treatment	Recruitment rates	Retention rates	
Huang et al., (2019) [36]	Intervention *n* = 27; mean: 61 Control *n* = 28; mean 58.68	Taiwan		Exercise Group: IIIA: 0; IIIB: 6; IV: 21 Control Group: IIIA: 1; IIIB: 2; IV: 23 Treatment: Chemotherapy	91.67%	100%	
Park et al., (2019) [34]	*n* = 100; mean: 55.1	South Korea		Stage: II: *n* = 5; III: *n* = 0; IV: *n* = 95 Treatment: Chemotherapy	No Information	90% (90/100)	
Ji et al*.,* (2019) [33]	Fixed-Interactive Exercise Group *n* = 32; mean 60.50 Fixed Exercise Group *n* = 32; mean 57.97	South Korea		Fixed-Interactive Exercise Group Stage: I: *n* = 13; II: *n* = 3; IIIA: *n* = 6; IIIB: *n* = 1; IV: *n* = 8 Fixed Exercise Group Stage: I: *n* = 7; II: *n* = 5; IIIA: *n* = 7; IIIB: *n* = 0; IV: *n* = 13 No Treatment Information	40.5% (64/158)	Fixed Exercise Group: (23/32): 71.88% Fixed-Interactive Exercise Group Stage: (20/32): 86.96% Total (43/64): 67.19%	
Lafaro et al., (2019) [37]	Intervention group *n* = 18; median: 74	USA		ASA: III: *n* = 12; IV: *n* = 2; V: *n* = 1 No Treatment Information	86.96% (20/23)	90% (18/20)	
Timmerman et al., (2017) [13]	Intervention group: Stage 1 *n* = 10; median 56.6 Stage 2 *n* = 12; median 59.5	Amsterdam	Disease Stage not reported Stage 1 Treatment: Lobectomy: n = 10; Pneumectomy: n = 0; Neoadjuvant: n = 1; Adjuvant: n = 3 Stage 2 Treatment: Lobectomy: *n* = 8; Pneumectomy: *n* = 2; Neoadjuvant: *n* = 2; Adjuvant *n* = 1		Consent Rate: 67%		67% (8/12)
Coats et al., (2019) [35]	Intervention group *n* = 5; mean 62	Canada		Stage 3 IIIb = 3; Stage IVb = 2; Treatment: Chemotherapy	No Information	100% (5/5)	
Maguire et al., (2015) [15]	Intervention group *n* = 16; mean: 63.6	United Kingdom		No Disease Stage Information Treatment: thoracic radiotherapy	28.1%	5 died (11/16) 68.75%	
Denis et al., (2014) [32]	Intervention group *n* = 42; median: 62	France		Stage: I/II: *n* = 9; IIIA: *n* = 15; IIIB: *n* = 1; IV: *n* = 17 Current Treatment: None 36; Maintenance therapy 6 Previous treatment: Surgery 11; Radiotherapy 1; Concomitant radio-chemotherapy 13; Chemotherapy 17	Not reported	Two died (95.24%)	

*USA*, United States of America; *ASA*, American Society of Anaesthesiologists

### Intervention characteristics

The three primary domains explored within the eight studies were education (*n* = 1) [36], physical activity and exercise [13, 35, 37] (*n* = 3), and self-evaluation and symptom monitoring (*n* = 2) [15, 32]. Two studies of the eight combined exercise and symptom management (*n* = 2) [33, 34].

One study focused on investigating the impact of a web-based health education program on global quality of life, quality of life-related function, and symptom distress over a 3-month period [36]. Another 3-month intervention explored the outcomes of home-based pulmonary rehabilitation (PR) regarding exercise capacity, dyspnea symptoms, and QoL in adult receiving treatment for NSCLC [34]. One of the interventions explored the use of tele-health in two mediums: ambulant symptom and physical activity monitoring (S&PAM) and a web-accessible home-based exercise program (WEP) [13].

The majority of the supportive care was delivered via mobile phone-based applications (*n* = 4) [13, 15, 33, 34], with other mediums including websites (*n* = 1) [36], web-based applications (*n* = 1) [32], video conferencing (*n* = 1) [37], and a Tele-Rehab Station (*n* = 1) [35]. The Tele-Rehab Station consisted of an all-in-one computer system running on a Windows 8 interface. The computer station, developed by the Centre for Interdisciplinary Research in Rehabilitation and Social Integration in Quebec City, was equipped with bio-mechanical and physiological sensors and equipment. The system supports videoconferencing via a connected webcam, providing a medium to deliver the audio-visual communication.

Three studies specified the use of theories and models to inform the design and development [15, 36, 37]. The theories used were as follows: Lafaro et al. (2019) used the Chronic care self-management model (CCM) [38] [37], Huang et al. (2019) is based on Symptom Management theory (SMT) [39] and the e-learning theory [40] [36], and Maguire et al. (2015) used the Medical Research Council (MRC) Complex Interventions Framework [41] [15].

### Feasibility and acceptability

Of the eight studies, six were deemed feasible by the study authors [13, 15, 32, 34, 35, 37], with two studies not stating feasibility outcomes [33, 36]. Though, studies that did not explicitly state feasibility outcomes still presented recruitment and retention rates. Huang et al. (2019) reported 91.67% recruitment rate and 100% retention rate. Ji et al., (2019) reported 40.5% recruitment rate and 67.17% retention rate. Three studies did not report the recruitment rates [32, 34, 35]. The mean recruitment rate of the five studies was 62.83 ± 27.99% [13, 15, 33, 36, 37]. Only one study reported a recruitment goal, which was not met [15].

The mean retention rate for the eight studies was 84.77% (67–100%). In two studies, the loss to follow-up was due to the death of participants during the studies [15, 32]. Several concerns pertaining to recruitment were noted, such as little or lack of familiarity with digital technology and the internet, emotional burden, poor health status, lack of interest, knee replacement, scheduled surgery, and patients felt adequately supported by their clinical team and required no further supportive care [13, 15, 34, 37]. Reasons noted for dropout were emotional burden, complications following surgery, cancellation of surgery, and hospital transfer [13, 34, 37]. Of the eight studies, none reported cost or financial cost of the study. Majority of the studies require health care professionals, researchers, and equipment, yet the monetary costs were not discussed. One study highlighted the absence of costing the intervention as a limitation [13]. Detailed information on feasibility results can be found in Table 3.

Due to the varying study designs, adherence was assessed in only three studies [32, 35, 37]. Adherence rates and compliance rates were used as the two primary methods of assessing adherence within the given studies. The mean “adherence rate” was 84.5% (73.5–100%) in the three studies. Adherence rates were defined by the completion of forms [32], completion of exercise sessions [35], and mean sum of pedometer use preoperative and post-discharge [37]. Lafaro et al. (2019) presented adherence rate for both lung and gastrointestinal cancer combined, not as independent outcomes.

Five studies reported one or more measures of satisfaction, with majority of participants reporting they were highly satisfied with the interventions [13, 33–35, 37]. Three studies did not report measures of satisfaction [15, 32, 36]. One study reported that majority of participants felt reassured and the advice from the intervention was user friendly and easy to understand [15]. Of those which reported measures of satisfaction, two studies reported reasons for dissatisfaction. Reasons reported in one study were lack of interaction with health care professional, insufficient tailoring of exercises, inadequate insight into progression, and difficulty accessing via mobile phone [13]. The second reported dissatisfaction was due the occurrence of system errors and difficulty in handling the application [34]. No study reported any adverse effects throughout the study duration. Detailed information of the acceptability results can be found in Table 4.

**Table 4:** Intervention overview, engagement, and acceptability outcomes

Source (author and year)	Objectives and description	Engagement	Acceptability (satisfaction)	Conclusion
Huang et al., *(*2019) [36]	Objective: evaluate the effects of a web-based health education program on global QoL, QoL-related function, and symptom distress in patients diagnosed with advanced NSCLC The experimental group participated in the web-based education program twice a month for three months	Those who consented (55/60) completed all assessments	Satisfaction measures not discussed	The web-based program can improve global QoL, emotional function, and reduce top ten significant symptom distresses within the first three months post diagnosis and treatment of advanced-stage NSCLC patients Web-based health education can enhance self-learning to assist with coping with cancer, treatments, and side effects
Ji et al., (2019) [33]	Objective: explore the outcome of home-based pulmonary rehabilitation (PR) regarding exercise capacity, dyspnea symptoms, and QoL in patients being treated for NSCLC Participants were randomly allocated to a fixed exercise group or a fixed-interactive exercise group The fixed exercise group used only the fixed exercise program during the 12 weeks. The fixed-interactive exercise group received the app with the fixed exercise regimen for the first six weeks. Switching to an app with an interactive exercise regimen for the remaining six weeks	64 participants allocated to the two groups 49 made it to six weeks analysis; 43 made it to the 12 weeks analysis	Participant Satisfaction (Patient Global Assessment [PGA]): Week 6: *n* = 39, Mean (SD): 13.769 (3.681 Week 12: *n* = 39; Mean (SD): 15.077 (3.989)	Personalized mHealth PR can supplement traditional health care rehabilitation programs for NSCLC patients. Findings support the use of this technology to improve exercise capacity, dyspnea symptoms, and QoL
Denis et al., (2014) [32]	Objective: investigate whether patient self-evaluated symptoms transmitted via the internet could be used between pre-planned visits to indicate early disease relapse in lung cancer Patients report their weight and ten symptoms, such as appetite loss (anorexia), fatigue (asthenia), pain, cough, and breathlessness (dyspnea) weekly The physician would be notified via email when self-evaluated symptoms met a pre-specified criterion	564/691 of all forms were completed, which is 82% of the maximum Mean monthly compliance was 94% Mean weekly compliance was 79%	100% of participants felt reassured they were being followed by their oncologist	A weekly follow-up system using the internet deemed feasible to detect relapse or tumor progression with a high rate of compliance
Coats et al., (2019) [35]	Objective: investigate the feasibility, adherence, satisfaction, and technical issues of a home-based telerehabilitation intervention for patients with unresectable thoracic neoplasia receiving chemotherapy The intervention was an eight-week home-based telerehabilitation program (three sessions of ~ 75 min per week) using the eChez-Soi telerehabilitation platform. The platform provided a combination of interactive exercises with real-time physiological parameter acquisition Sessions started off supervised but with study progression reduced to mainly unsupervised	The mean duration of supervised sessions was 67 ± 12 min. Total duration of all 75 supervised exercises sessions was 85 h. Mean time for cardiovascular exercise was 247 ± 48 min over the 15 supervised exercise sessions and 223 ± 111 min over the 8.6 ± 3.0 unsupervised exercise sessions. Mean duration of each cardiovascular exercise session was 18 ± 6 min and 26 ± 9 min during supervised and unsupervised exercise sessions	5/5 patients reported being quite satisfied (score of 4) or very satisfied (score of 5) with all aspects of the home-based telerehabilitation platform. Mean satisfaction score: 4.7 ± 0.4	Findings support the feasibility of a Tele Rehabilitation program (TELERP) and suggest the intervention may help patients overcome barriers to pulmonary rehabilitation services Participation in TELERP may assist improvements or maintenance in muscle strength and functional capacity for lung cancer patients on chemotherapy treatment
Timmerman et al., (2017) [13]	Objective: evaluate the feasibility of a Tele-healthcare application for operable lung cancer patients Stage One: Prior to the start of the study, thoracic surgeons and pulmonologists were given a short presentation about content and possible benefits of the symptom and physical activity monitoring S&PAM module Physiotherapists were introduced to the web-accessible exercise program (WEP) during a two-hour workshop Stage Two: The Remote Monitoring and Treatment RMT it consists of two modules: (1) a symptom and physical activity monitoring (S&PAM) system, and (2) a web-accessible exercise program with remote supervision by a physiotherapist	Ambulant S&PAM system: 100% of patients used the S&PAM system at least once Mean usage: Five—six days per treatment period WEP: Eight patients (67%) used the exercise portal at least 1 week following lung resection. Patients started 4 (n = 3), 5 (n = 2), 6 (n = 2), or 7 (n = 1) weeks following resection	S&PAM: most patients indicated that the monitoring system had good usability. All felt competent using the module (perceived self-efficacy score > 5) WEP: most patients were satisfied with usability of the module, except for two (score < 5) stating the program was difficult to access on mobile phone All patients felt confident in their ability to use the module	Findings support that remote monitoring and treatment is feasible to lung cancer patients both pre- and post-surgery Patients actively used the S&PAM and WEP modules prior and following surgery and perceived both as a beneficial contribution to their care A low level of adoption by referring physicians may reduce successful implementation
Lafaro et al., (2019) [37]	Objectives: (1) determine the feasibility and acceptability of a personalized telehealth intervention, for physical activity perioperatively for GI and lung cancer patients and their caregivers. (2) describe the trends, trajectories, and patterns of both functional recovery and self-reported outcomes pre- and post-surgery The intervention consisted of five sessions. Session one was after baseline assessment and a minimum of seven—fourteen days prior to surgery via videoconferencing. Session two (in-person) functional re-assessment (6MWT, TUG, SPPB) and self-reported measures. Session two content was delivered post re-assessment. Sessions three, four, and five (telehealth) were completed at days two, seven, and two—four weeks post-discharge. All given outcomes were re-assessed at two—four weeks post-discharge. Acceptability was measured via a satisfaction survey. Pedometer data was collected throughout the study duration Strategies to overcome barriers to staying active after discharge were discussed	Preoperative pedometer adherence: 79%, post-discharge 68%. Median preoperative daily steps were 6324 The value decreased to 1050 during hospitalization, The value increased to 2927 in the first 2 weeks after discharge	Self-reported satisfaction: 3.2/4.0 93.3% of patients thought that the timing of the intervention was appropriate	The personalized telehealth perioperative physical activity intervention was feasible and acceptable for both adults undergoing GI or lung cancer surgery and their caregivers
Park et al. (2019) [34]	Objective: determine the feasibility and efficacy of smartphone app–based PR on QoL, exercise capacity, and symptom management for patients with advanced lung cancer who were undergoing chemotherapy Patients were provided with the Smart Aftercare app, an Internet of Things (IoT) wearable device, a portable pulse oximeter, thermometer, scale, and resistance bands. The to-do list provided an alarm notification for daily tasks related to taking medication, performing rehabilitation exercise, and visiting the clinic on schedule This study consisted of a 12-week rehabilitation program. The Smart Aftercare app provided animation videos on stretching exercises, aerobic exercises, muscle strengthening exercises, and finishing (stretching) exercises. The Smart Aftercare app provided an animation video on pain control, nutritional support, and symptom management	90 finished the rehab program 85/90 completed all 6MWT tests	Satisfaction: 77% (69/90) reported they were satisfied 88% (79/90) reported they would recommend it to others 96% (86/90) stated they were paying more attention to their health and disease status since using the app	12 weeks of comprehensive smartphone app–based individualized PR seems to be an effective and feasible approach for improving exercise capacity, symptom management, and distress in patients with advanced NSCLC undergoing systemic chemotherapy
Maguire et al. (2015) [15]	Objective: (a) explore the feasibility and acceptability of the Advanced Symptom Management System with patients with lung cancer receiving radiotherapy (ASyMS-R) and clinicians involved in their care; (b) assess changes in patient outcomes during implementation of the ASyMS-R in clinical practice Patients used the ASyMS-R at home during working hours (9 AM—5 PM), seven days a week, for the duration of their radiotherapy treatment in addition to one month after treatment. They were instructed to follow local procedures regarding out-of-hours care After completing the daily questionnaire on their mobile phone, patient’s daily symptom data were sent to a central study server, where an integrated risk model analysed the symptom reports	89% of participants reported the self-care system was easy to understand and user friendly	Nine patients indicated that they had received enough training to use the ASyMS-R handset 100% of patients reported that they never or very rarely encountered problems in using the handset (n = 10; 100%), answering and submitting questionnaires (n = 9; 90%), reading the self-care information after submitting a questionnaire or again later (n = 10; 100%), or finding cancer information pages (n = 8; 89%)	This study demonstrated the potential to provide an accurate and acceptable assessment of radiotherapy-related toxicity and management in clinical practice. Therefore, effectively responding to the needs of patients in this study and facilitating the delivery of timely interventions. Participants reported the ASyMS-R to positively impact on their care and promote the timely reporting and management of their symptoms

*QoL*, Quality of Life; *NSCLC*, Non-Small Cell Lung Cancer; *PR*, Pulmonary Rehabilitation; mHealth, Mobile Health; *SD*, Standard Deviation; *TELERP*, Tele Rehabilitation program; *WEP*, web-accessible exercise program; *RMT*, Remote Monitoring and Treatment; *S&PAM*, symptom and physical activity monitoring; *GI*, Gastrointestinal; *6MWT*, 6 min walk test; *TUG*, Time Up and Go; *SPPB*, Short Physical Performance Battery; *IoT*, Internet of Things; *ASyMS-R*, Advanced Symptom Management System with patients with lung cancer receiving radiotherapy; *PGA*, Patient Global Assessment

### Efficacy

Efficacy outcomes are only reported for RCTs. Of the eight studies, there were two RCTs [33, 36]. Outcomes assessed included QoL, physical functioning [33], and symptom distress [36].

#### Quality of life

Participants who participated in an online-based health education program had a significant increase in global QoL in comparison to a control group [36]. All participants who participated in a mobile-based pulmonary rehabilitation platform exhibited an overall significant increase in QoL (visit one, 76.05 ± 12.37; visit three, 82.09 ± 13.67 (*P* = 0.002)), assessed using a visual scale (EuroQol-visual analog scale). However, a small, non-significant change in QoL was observed (visit one, 7.535 ± 1.817; visit three, 6.930 ± 2.849 (*P* = 0.17)) via the EQ-5D questionnaire (EuroQol 5 dimensions questionnaire) [33]. There was not a significant difference pre-intervention and post-intervention for QoL between the Fixed-interactive exercise group and Fixed exercise group for both visual scale (*P* = 0.99) or EQ-5D (*P* = 0.50) [33].

#### Emotional functioning

Participants who engaged in the online health education program reported significant improvements in emotional function in comparison to those who did not [36]. In fact, those who did not engage with the online health education program displayed a non-significant decrease in emotional function [36]. Significance was determined from baseline (T0) to three months after the program (T3) for both experimental and control groups via the European Organization for Research and Treatment of Cancer Quality of Life Questionnaire Core 30 (EORTC QLQ-C30).

#### Physical functioning

Participants who performed physical activity displayed an improvement in their physical function, assessed via their six minute walk distance (6MWD) over a 12-week period (visit one, 433.429 ± 65.595; visit three 471.250 ± 75.691 (*P* = 0.001)). However, no statistical significant difference (*P* = 0.30) was reported between the fixed exercise group (58.095 ± 73.663) and fixed-interactive exercise group (25.368 ± 66.640) [33].

#### Symptom distress

Participants who participated in an online education program had a significant reduction (*P* < 0.05) in the top ten significant symptom distresses from baseline (1.45 ± 0.08) to three months post program (1.26 ± 0.06), whereas the control group demonstrated a non-significant increase (*P* = 0.530) from baseline (1.41 ± 0.09) to post three months (1.73 ± 0.27) [36]. Data on symptom distress was collected via the symptom distress scale.

## Discussion

This review aimed to examine the evidence of the feasibility, acceptability, and potential efficacy of online supportive care interventions for those LWBLC. The results show that online delivery of supportive care for people LWBLC is feasible and acceptable. However, the field of delivering supportive care in this population is in its infancy. To our knowledge, this systematic review is the first to explore feasibility, acceptability, and efficacy of online supportive care for people LWBLC.

Eight studies met the inclusion criteria, two of which were RCTs. The average recruitment rate was 62.58%, though this was not universally reported, and the average retention rate was 84.77%. Problems with recruitment and attrition are common among studies involving people living with and beyond cancer, especially people LWBLC [8]. The challenge recruiting people LWBLC stems from the high symptom burden and lower health performance status [8, 42]. Low rates of participation and consent are common among people living with and beyond cancer, people with advanced diseases, and those approaching palliative end of life care [43, 44]. Older adults (≥ 65y) are reported to be underrepresented in research, with a small increase of older adults in oncological clinical trials over the recent years [45]. Though, people LWBLC typically tend to be older individuals, with 44% of new diagnosis of lung cancer in the UK among those 75 years or older [46], yet the mean age for the included studies was 61 years. This affirms the aforementioned argument by Hurria et al. (2014) that older individuals are underrepresented in oncological research, suggesting that consideration should be given when interpreting the results for this population. The capabilities of older adults to use digital technology is often questioned within literature [34], although elderly adults are becoming increasingly literate using digital technology and eager to adopt new technologies [47].

Adding to the growing body of literature exploring the use of online supportive care for people living with and beyond cancer, this review shows emerging evidence that online supportive care platforms are also feasible and acceptable for people LWBLC. This aligns with the larger body of literature among breast [48, 49], prostate [16, 50], colorectal cancer [51, 52], and chronic obstructive pulmonary disease (COPD) [53, 54], a progressive chronic lung disease which has similar symptoms and QoL impact to lung cancer [55]. This evidence suggests that online supportive care is feasible and acceptable in these populations.

Engaging in supportive cancer care is important for management of symptoms and improvements in quality of life for people LWBLC [8]. In the current global Coronavirus Disease (COVID-19) pandemic, people living with and beyond cancer are at greater risk of experiencing serious illness if tested positive for the COVID-19 pandemic [56], particularly those receiving chemotherapy and/or radiotherapy for lung cancer [57]. Throughout the pandemic, the frequency of in-person assessments and programs have been severely reduced, leading to a variety of concerns such as, missed diagnosis, unnoticed development of new symptoms, unobserved disease progression, reduction in physical activity sessions, and access to educational resources. Literature has reported weekly symptom monitoring via a web-based patient-reported outcomes platform that was associated with increase survival for those living with and beyond metastatic cancer compared to standard care [58] and those LWBLC in comparison to standard imaging surveillance [59]. Therefore, the importance of delivering supportive care via online modalities is paramount. However, even before the COVID-19 pandemic, barriers existed supporting the implementation of any supportive care for people LWBLC. Economically, there is a considerable financial burden associated with lung cancer, both societal and personal [60]. The cost of travel is an out-of-pocket expensive which could be a barrier for people living with and beyond cancer to access appointments and treatments [60]. In addition, various studies have associated lower socioeconomic status (SES) with higher incidence of lung cancer [61, 62]. The use of digital technology and telehealth has become more prevalent since the COVID-19 pandemic [63], with an exponential growth in platforms such as videoconferencing [57], although the evidence pertaining to online supportive care for people LWBLC is still limited. The evidence that lung cancer is overshadowed in the literature by other forms of cancer is clear within both supportive care in both standard and online modalities [64, 65]. With the complexity of the current global climate, many individuals are unable to seek the supportive care usually provided. This systematic review provided a timely contribution to the sparse knowledge of online supportive care for people LWBLC.

To advance this area, more rigorous research must be conducted, building upon the available pilot-based studies, such as ensuring adequately powered samples and generalisability of results [66]. The studies conducted have shown to have a lower mean age than that of the average for a lung cancer diagnosis. Furthermore, RCTs using a clear randomisation process should be performed to explore the effects online supportive care can present in comparison to well-balance groups [67]. Conducting trials over multiple sites may prove useful regarding greater samples for recruitment. Furthermore, literature suggests that methodological appraisal is often misapplied when assessing non-randomised studies [26]. Studies must appropriately appraise methodological quality of their literature to provide high quality evidence.

## Conclusion

Online supportive care for people living with and beyond cancer has shown promise within this review. Given the complexity of delivering cancer services online, the current global COVID-19 pandemic has highlighted the need for online supportive care for people living with and beyond cancer, specifically lung cancer [57]. The studies discussed in this review cover two primary domains of supportive care, symptom management, and increasing QoL, which have been highlighted as key components of supportive care [8]. This illustrates that key components of supportive care can be administered online, showing feasibility and acceptability. Though, the concept of adherence rates requires further exploration within this population. A recent shift has been observed from inpatient to ambulatory care for people living with and beyond cancer and an increased number of outpatients receiving treatment has rapidly increased [15] leading to more individuals being responsible for self-management of treatment-related toxicities within their own home. The use of digital technology such as mobile or web-based platforms to enable real-time communications could be vital in supportive care.

This review provides evidence that online supportive care programs for people LWBLC are feasible and acceptable. The conclusions are limited to a small number of studies, though the strong methodological quality of the studies provide strength in the results. With limited evidence presented from RCTs, it is difficult to determine efficacy. Though online supportive care within lung cancer is in its infancy, further larger RCTS and rigorous studies are warranted.

## Supplementary Information

Below is the link to the electronic supplementary material.

Supplementary file1 (PDF 35.2 KB)

## Data Availability

Not applicable.
