# Supplementary material for: Feasibility, acceptability, and efficacy of online supportive care for individuals living with and beyond lung cancer: a systematic review
Source: Support Care Cancer. 2021 May 18;29(11):6995–7011. doi: 10.1007/s00520-021-06274-x (PMC8130779; doi:10.1007/s00520-021-06274-x)
Supplement: Supplementary file 1 — Supplementary file1 (PDF 35.2 KB) [file 520_2021_6274_MOESM1_ESM.pdf]

Supplemental Material

Title: Feasibility, acceptability, and efficacy of online supportive care for individuals living with and beyond lung cancer: a systematic review.

Journal Name: Supportive Care in Cancer

Authors:

Jordan Curry[a], Michael Patterson[a], Sarah Greenley[b], Mark Pearson[a], Cynthia C. Forbes[a]

Affiliations:

[a]Wolfson Palliative Care Research Centre, Hull York Medical School, University of Hull, Hull, United Kingdom.

[b]Institute for Clinical and Applied Health Research, Hull York Medical School, University of Hull, Hull, United Kingdom

Corresponding Author Email:

Jordan.Curry@hyms.ac.uk.

Appendix 1: Search strategy for Medline via OVID

1. exp Lung Neoplasms/
2. (Lung adj2 (cancer* or neoplasm* or tumor* or tumour* or carcinoma*)).ti,ab,kw.
3. exp Cancer Survivors/
4. or/1-3 [Lung Cancer Concepts]
5. exp internet/ or exp internet access/ or social media/ or exp Cell Phone,/ or Smartphone/
6. Online Care.ab,ti,kw.
7. Online Support.ab,ti,kw.
8. eHealth.ab,ti,kw.
9. mHealth.ab,ti,kw.

10. Mobile Phone*.ab,ti,kw.

11. Smart Phone*.ab,ti,kw.

12. Smartphone*.ab,ti,kw.

13. Internet*.ab,ti,kw.

14. Mobile App*.ab,ti,kw.

15. Mobile Application*.ab,ti,kw.

16. Website*.ab,ti,kw.

17. eSupport.ab,ti,kw.

18. Digital Support.ab,ti,kw.

19. Web* Support.ab,ti,kw.

20. Telehealth.ab,ti,kw.

21. patient portal.ab,ti,kw.

22. exp Telemedicine/

23. or/5-22 [Internet Concepts]

24. ((message or discussion) adj3 (board* or internet or online)).ti,ab,kw.

25. (chatroom* or (chat adj room)).ti,ab,kw.

26. ((online or discussion) adj3 forum*).ti,ab,kw.

27. (social media or Facebook or Twitter or Instagram or blog* or YouTube or WhatsApp).ti,ab,kw.

28. exp Blogging/

29. or/24-28 [Social Media and Online Discussions Concepts]

30. 23 or 29

31. 4 and 30
